# Initiator Systems Effect on Particle Coagulation and Particle Size Distribution in One-Step Emulsion Polymerization of Styrene

**DOI:** 10.3390/polym8020055

**Published:** 2016-02-19

**Authors:** Baijun Liu, Yajun Wang, Mingyao Zhang, Huixuan Zhang

**Affiliations:** 1 School of Chemical Engineering, Changchun University of Technology, Changchun 130012, China; liubaijun111@126.com (B.L.); yjwang_bio@163.com (Y.W.); zhanghx@mail.ccut.edu.cn (H.Z.); 2 Changchun Institute of Applied Chemistry, Chinese Academy of Sciences, Changchun 130022, China

**Keywords:** emulsion polymerization, particle size distribution, coagulation, initiator

## Abstract

Particle coagulation is a facile approach to produce large-scale polymer latex particles. This approach has been widely used in academic and industrial research owing to its higher polymerization rate and one-step polymerization process. Our work was motivated to control the extent (or time) of particle coagulation. Depending on reaction parameters, particle coagulation is also able to produce narrowly dispersed latex particles. In this study, a series of experiments were performed to investigate the role of the initiator system in determining particle coagulation and particle size distribution. Under the optimal initiation conditions, such as cationic initiator systems or higher reaction temperature, the time of particle coagulation would be advanced to particle nucleation period, leading to the narrowly dispersed polymer latex particles. By using a combination of the Smoluchowski equation and the electrostatic stability theory, the relationship between the particle size distribution and particle coagulation was established: the earlier the particle coagulation, the narrower the particle size distribution, while the larger the extent of particle coagulation, the larger the average particle size. Combined with the results of previous studies, a systematic method controlling the particle size distribution in the presence of particle coagulation was developed.

## 1. Introduction

Emulsion polymerization is a widely used process for the production of rubber, plastic, coating, and adhesives in industry [1,2,3,4,5]. Control over size and polydispersity in these applications is required because of the close relationship between the properties of the polymer and the particle size distribution [6,7,8,9,10,11,12,13]. Thus, how to control particle size and polydispersity has gradually become an essential issue. Until today, many technologies based on emulsion polymerization including seeded emulsion polymerization and agglomeration method [1,3,6,10,14,15,16] have been proposed to control the particle size and distribution. Among these technologies, the particle coagulation technology has been accepted as a highly effective approach to prepare nanoparticles in both industrial production and theoretical investigation [1,7,15,17,18,19].

**Scheme 1:**
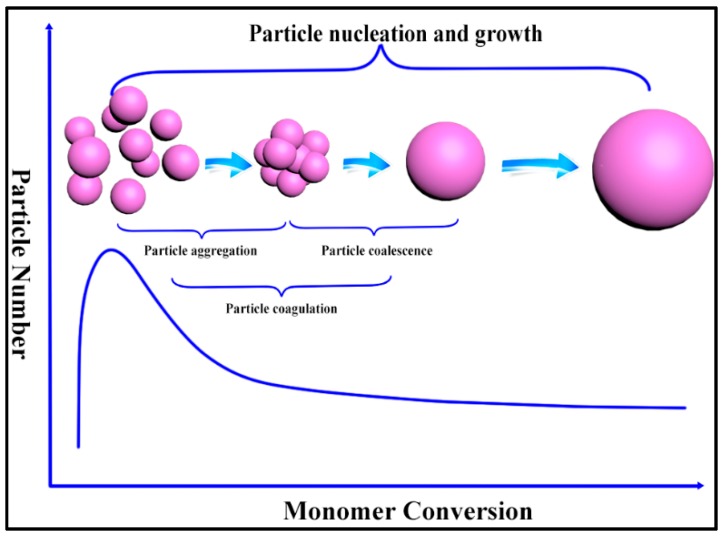
The schematic diagram of particle coagulation in one-step emulsion polymerization.

Particle coagulation is a process that occurs in the period of the particle nucleation and growth. Even though it is induced by the increase in the interfacial energy change, it is also a kinetically controlled process. Particle coagulation can be divided into two periods, as shown in Scheme 1: (1) the process of the particle aggregation is determined by the probability of the particle collision. Some factors such as the viscosity of the media and the reaction temperature can directly affect this process [20]. In agreement with the Smoluchowski equation [21,22], the kinetic of the particle coagulation can be expressed by $-\frac{dN}{dt}=k_{c}N_{0}^{2}$, where $-\frac{dN}{dt}$ is the particle coagulation rate, and $k_{c}$ and $\;N_{0}$ are a constant and particle number, respectively. In another period, several particles merge into a larger one, which is determined by the particle structure and glass transition temperature [23,24]. The effect of simple reaction parameters on particle coagulation in emulsion polymerization using pure water as solvent has been well illustrated by many researchers. For instance, Dobrowolska *et al.* investigated the effect of ionic strength on the extent of particle coagulation and particle size distribution, and illustrated that higher ionic strength could decrease the thickness of the diffuse electric double layer, and further increase the extent of particle coagulation [17,18,25]. Chern *et al.* elaborated the role of surfactant systems in determining particle coagulation and found that the relationship between the particle number and the surfactant concentration [26,27].

As usual, the emulsion polymerization is carried out in water as the medium. However, recent investigations indicated that the addition of co-solvent to the medium played an important role in determining the particle size and distribution of the ultima latex. For example, Adelnia *et al.* investigated that the effect methanol on the characteristics of the Poly (methacrylate-*co*-butyl acrylate) latex, and found that the addition of methanol increased the medium viscosity and further facilitated the particle coagulation [20]. Kim *et al.* also carried out the investigation of soap-free emulsion polymerization of methyl methacrylate in different methanol solutions, and indicated that the polymerization product and behavior resembled those typical of pure water [28].

In our previous reports, we carried out a series of experimental investigations about particle coagulation, and stressed the role of a co-solvent such as methanol in determining the extent of the particle coagulation [29,30,31,32]. With the increase in methanol concentration in aqueous phase, the interfacial tension between the aqueous phase and particles decreased. This decreased the adsorption capability of the surfactant molecules on particle surface and further decreased the repulsive potential energy. On the other hand, the attractive potential increased because of the decrease in the Hamaker constant. As a result, the extent of the particle coagulation was enhanced. In addition, the addition of the methanol also increased the length of the critical chain length (CCL) when polymer chains precipitated from the aqueous phase, further increasing the initial particle size, and decreasing the polymerization reaction rate. Even though particle coagulation has been studied extensively, some fundamental issues such as the effect of the initiator systems on particle coagulation are still puzzling to investigators.

Radical polymerization initiator systems can be divided into many categories. From the solubility view, the initiator systems can be divided into water-soluble and oil-soluble initiators; from another view, it can also be divided into ionic and nonionic types. The ionic type initiator system is further divided into cationic and anionic types based on the difference in the charges. The effect of the initiator system on particle coagulation seems to be an easy problem, but it is indeed an extremely complicated phenomenon. According to the Smith–Ewart theory, pure water was usually chosen as solvent, and the particle number increased with increasing initiator concentration [3], but the Smoluchowski equation indicated that the rate of particle coagulation also increased with increasing particle number, as described above [21,22]. Thus, the addition of the initiator seems to play an opposite role in determining particle number, and what is its main function? The ionic initiator not only plays the role of the initiator, but also function as an electrolyte, which increases the complexity of the particle coagulation. In the case of opposite charged surfactant and initiator, does the shielding action between surfactant molecules adsorbed on particle surface and the initiator chain ends affect the extent of the particle coagulation? Can the time of particle coagulation be adjusted by initiator systems?

In regard to the effect of the oil-soluble initiator on particle nucleation mechanism in emulsion polymerization, two main mechanisms for the production of radicals were postulated. One of the mechanisms considered that the first radicals were generated in the monomer-swollen polymer particles/monomer droplets/monomer swollen micelles, and desorbed to the continuous phase; another mechanism considered that the radical formed in the continuous phase were derived from the fraction of the oil-soluble initiator dissolved in the continuous phase [33,34,35,36,37]. Nomura *et al.* indicated the relationship between the particle number (and molecular weight) and recipe compositions when oil-soluble initiator was used in pure water solvent. For example, Nomura *et al.* carried out the unseeded and seeded emulsion polymerization of styrene using azodiisobutyronitrile (AIBN) as an oil-soluble initiator and concluded that the latex particles were formed in the emulsifier micelles, and the particle number was proportional to the 0.30th power of the concentration of the initiator [38,39]. Capek *et al.* also reported that the kinetic aspects initiated by AIBN in the presence of a blend of anionic and non-ionic surfactant conditions and stressed that the addition of AIBN decreased the molecular weight and the polymerization reaction rate [40,41]. Recently, Gugliotta *et al.* investigated the role of the oil-soluble initiator in governing the particle nucleation in mini-emulsion polymerization, and indicated that the oil-soluble initiator could promote droplet nucleation and control the second nucleation [42]. Even though the kinetic model and experimental evidence of the emulsion polymerization initiated by oil-soluble initiator have been developed, it is still difficult to control the particle size distribution of ultima latex when oil-soluble initiator is used, especially to prepare narrowly dispersed polymer latex particles.

To address these problems, in this study, different initiator systems such as water-soluble potassium persulfate (an anionic type initiator), oil-soluble azodiisobutyronitrile (a nonionic type), and 2,2’-azobis [2-methylpropionamidine] dihydrochloride (AIBA) (a cationic ionic type) were chosen to initiate the polymerization reaction of styrene in the presence of methanol solution. The evolution of the particle size, number, and distribution as a function of polymerization time was traced for achieving a comprehensive understanding about particle coagulation. The polymerization condition selected here is based on the idea that obvious particle coagulation occurs, although, in many situations, particle coagulation is not obvious. The effect of reaction parameters except initiator systems on the particle coagulation and particle size distribution could be obtained from our previous studies [23,29,30,31,32].

## 2. Materials and Methods

Chemical Styrene (St, 99%), supplied by the Shanghai Chemical Reagent Corporation (Shanghai, China), was distilled under vacuum to remove the inhibitors prior to polymerization and used as the monomer. Sodium dodecyl sulfate (SDS; 99%; Aladdin, Shanghai, China) and Potassium carbonate (K_2_CO_3_; 98.5%; Aladdin, Shanghai, China) were used as the surfactant and electrolyte without any further purification. Potassium persulfate (KPS; 99.5%; Aladdin, Shanghai, China), Azodiisobutyronitrile (AIBN; 98%; Aladdin, Shanghai, China), 2,2’-azobis [2-methylpropionamidine] dihydrochloride (AIBA, 99%; Aladdin, Shanghai, China) were used as the initiator. Double distilled-deionized (DDI) water was used in all experiments. The polymerization fundamental recipe for investigating the effect of initiator systems on particle coagulation is shown in Table 1.

### 2.1. Polymerization Reactions

The emulsion polymerization reactions of styrene were carried out using a 500 mL glass reactor equipped with four necks for string mechanically with an anchor stirrer (Bar Length: 300 mm; Blade Diamete: 45 mm; Surface Coating Material: Polytetrafluoroethylene), condensing the reflux with cold water, purging with nitrogen gas, and sampling an aliquot of the solution with a pipette. The SDS, K_2_CO_3_, DDI and co-solvent (methanol) were added to reaction equipment according to this sequence; subsequently, monomer was also added when all auxiliaries were dissolved in the aqueous phase. Nitrogen (N_2_) purging was carried out for 30 min before the initiator was added to the reactor. When the initiator dissolved in some DDI or monomer was added into the equipment, the polymerization reaction begins. The polymerization reaction was carried out under the N_2_ atmosphere, the reaction temperature and stirring rate were set as 65 °C and 250 rpm, respectively. During the polymerization process, 2–3 g latex was withdrawn from the reactor using a syringe at appropriate intervals to analyze the monomer conversion, particle size distribution and particle number. The polymerization reaction time was set as 6 h.

### 2.2. Characterization

The particle size and distribution was measured by the Brookhaven 90plus Particle Size Analyzer (Brookhaven, NY, USA) and transmission electron microscopy (TEM) (JEOL 1210, Tokyo, Japan). The polydispersity index (PDI) was directly obtained from the Particle Size Analyzer, and defined by ISO 13321: 1996 E. The particle number (*N*_p_) was obtained by the following equation [32]:

(1) $$N_{p}=\frac{6XM_{0}}{\pi d_{p}^{3}\rho_{p}}$$

where *M*_0_ is the monomer concentration in the unit mass aqueous phase (1 kg for aqueous phase), $\rho_{p}$ is the polymer density, *X* is the monomer fractional conversion (which could be obtained by the gravimetric method) and *d_p_* is the average size of the latex particle.

## 3. Results

### 3.1. KPS Initiator System

**Figure 1:**
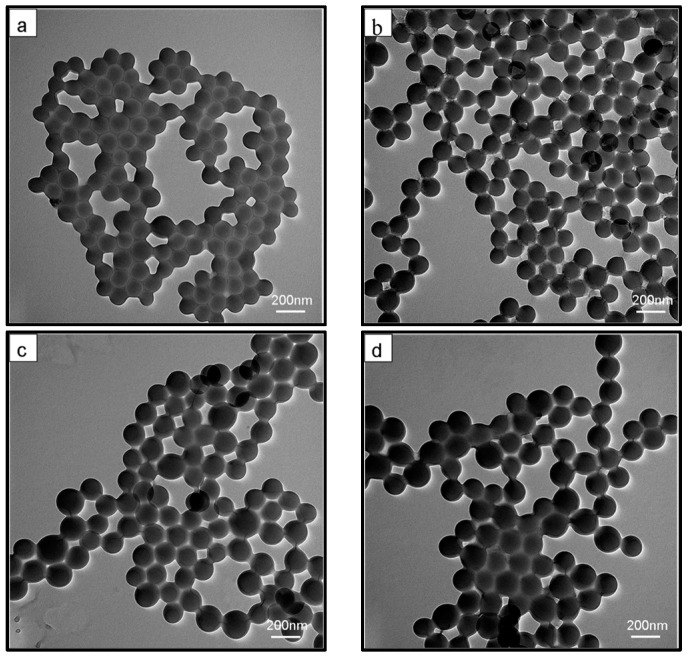
TEM micrographs of the final particles prepared by emulsion polymerization of styrene using methanol solution (20/80 *w*/*w*) as the polymerization medium and various amount of initiator KPS: (**a**) 0.3 wt %; (**b**) 0.6 wt %; (**c**) 0.9 wt %; and (**d**) 1.2 wt %.

To better understand the effect of the initiator system on the particle coagulation and particle size distribution, the KPS system was first considered. The KPS initiator system is one of the most widely used initiators in conventional emulsion polymerization in pure water solvent [3,10]. The theory of Smith–Ewart (micellar theory) predicted a proportionality value of 0.40 between particle number and KPS initiator concentration; Sajjadi *et al.* performed the emulsion polymerization of butyl acrylate using KPS as initiator according to the method of Capek [41], and found that the particle number was proportional to 0.39th power of KPS concentration [43]. These theories confirmed the relationship between the particle size and initiator concentration, indicated that the particle size of the ultimate latex decreased with increasing the initiator concentration. In this study, the particle size of the ultimate latex corresponding to different initiator concentrations is shown in Figure 1. The particle size increased with increasing initiator concentrations (97.9, 107.5, 136.7, and 161.5 nm at 0.3, 0.6, 0.9 and 1.2 wt %, respectively), and was in reverse trend to that of particle size in the conventional emulsion polymerization.

**Figure 2:**
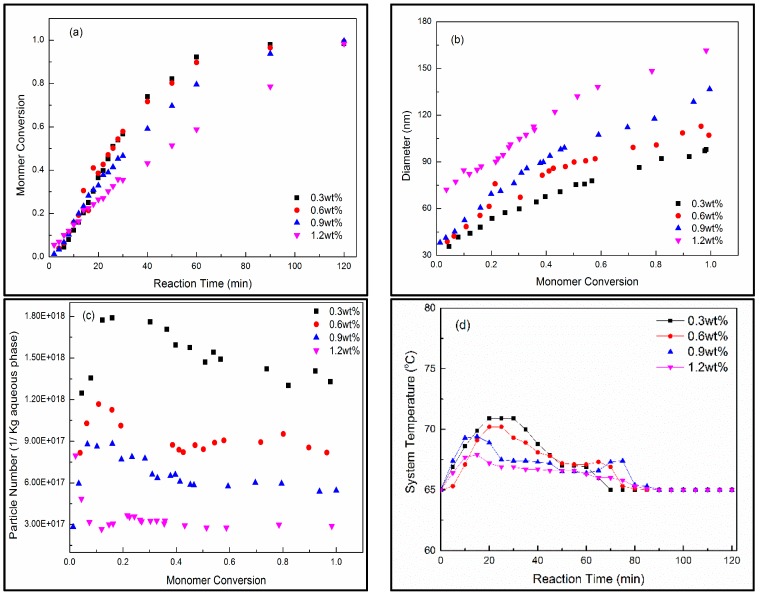
Plots of monomer conversion (**a**); particle size (**b**); number (**c**); and system temperature (**d**) against reaction time (or monomer conversion) for the emulsion polymerization of styrene using methanol solution (20/80 *w*/*w*) as the polymerization medium and various amount of initiator KPS ([*I*] = 0.3, 0.6, 0.9 and 1.2 wt %).

In addition, the evolution of monomer conversion, particle size and number as a function of the polymerization process for different initiator concentrations is traced, as shown in Figure 2. The curve of monomer conversion *vs.* the polymerization time, as shown in Figure 2a, indicates that the polymerization rate using 0.3 wt % KPS initiator system was the fastest among these reactions and was opposite to the results reported by Carro *et al* [21]. Figure 2b shows that the particle size rapidly reached 90 nm at 0.2 conversion in the presence of 1.2 wt % KPS. Meanwhile, the particle size corresponding to the systems consisting of 0.3, 0.6, and 0.9 wt % KPS only attained 54.2, 62.1, and 70.0 nm, respectively. Figure 2c shows that the particle number increased with increasing conversion, then decreased until reaching a constant particle number at appropriate conversion. The decrease in particle number and the increase in particle size indicated that the particle coagulation occurred during the polymerization process [29,30,31,32]. With increasing initiator concentration, the extent of particle coagulation gradually increased and the time of particle coagulation advanced. In addition, the evolution of the system temperature inside reactor also reflects some information about the particle coagulation, as shown in Figure 2d. The increase in the system temperature was mainly attributed to the heat released from the system larger than the heat transmission from the reaction system to the environment [44,45]. The increase in temperature was also considered as an evaluated parameter to indicate the polymerization reaction rate [46]. Figure 2d shows that the maximum system temperature decreased with increasing initiator concentrations from 71.2, 70.2, and 69.1 °C at 0.3, 0.6 and 0.9 wt % KPS, respectively, to 67.9 °C at 1.2 wt %. Moreover, the time corresponding to the maximum system temperature also advanced as the initiator KPS concentrations increased. With increasing initiator concentrations, the initial reaction rate was enhanced, and the particle number also increased. However, by increasing the particle number, the extent and time of the particle coagulation were enhanced. This decreased the rate of the polymerization reaction and led to the decrease of the system temperature [20].

**Figure 3:**
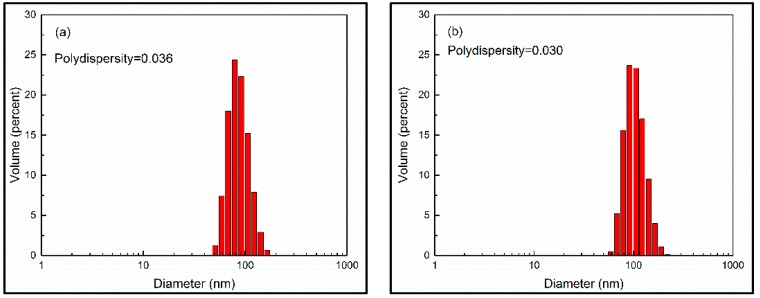

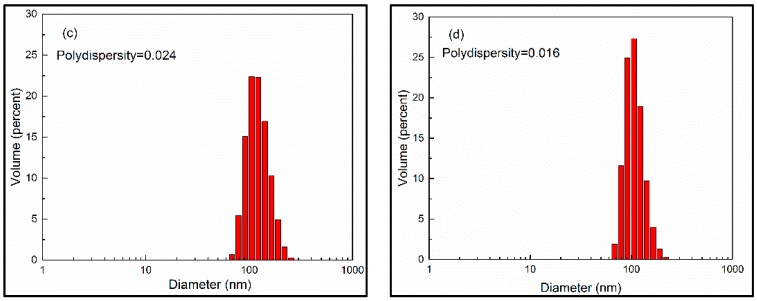
Particle size distribution of final latex particles prepared by emulsion polymerization of styrene using methanol solution (20/80 *w*/*w*) as the polymerization medium and various amount of initiator KPS: (**a**) 0.3 wt %; (**b**) 0.6 wt %; (**c**) 0.9 wt %; and (**d**) 1.2 wt %.

As a result of early particle coagulation, the width of particle size distribution of the final latex particles decreased with increasing initiator concentrations, as shown in Figure 3. The polydispersity index of ultimate latex particles decreased from 0.036, 0.030, and 0.024 at 0.3, 0.6, and 0.9 wt % KPS, respectively, to 0.016 at 1.2 wt % KPS. The polydispersity index of ≤0.1 indicates that the ultimate latex particles had a relatively narrow particle size distribution [47]. Meanwhile, the particles obtained by particle coagulation were spherical and smooth, indicating that narrowly dispersed latex particles could be prepared in the presence of particle coagulation.

**Figure 4:**
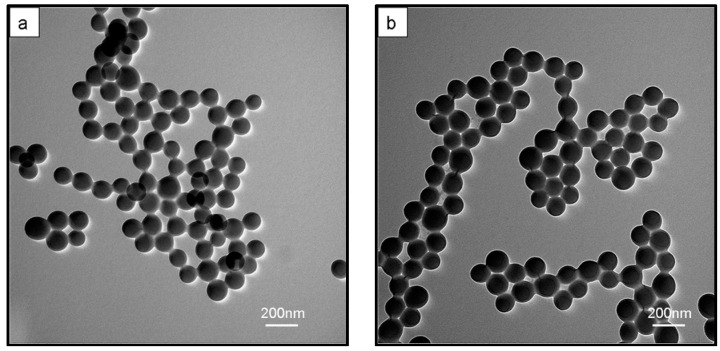
TEM micrographs of the final particles prepared by emulsion polymerization of styrene using methanol solution (20/80 *w*/*w*) as the polymerization medium and various reaction temperatures: (**a**) 55 °C and (**b**) 75 °C. The TEM micrograph of the final particles prepared by emulsion polymerization of styrene at 65 °C is shown in Figure 1b.

To ensure the role of increasing initiator concentration, the polymerization reactions with 0.6 wt % KPS initiator were carried out at the initiation reaction temperatures of 55 and 75 °C. The initiator decomposition rate is well known to increase with increasing reaction temperature [48]. Therefore, increasing initiation temperature also increased the primary radicals, thus increasing the extent of particle coagulation [45,46,48]. The TEM images of the ultimate latex particles prepared at different reaction temperature are shown in Figure 4. As expected, the average size of latex particles increased with increasing reaction temperature from 91.2 to 111.2 nm. Moreover, the polydispersity index value decreased from 0.031 at 55 °C to 0.015 at 75 °C.

**Figure 5:**
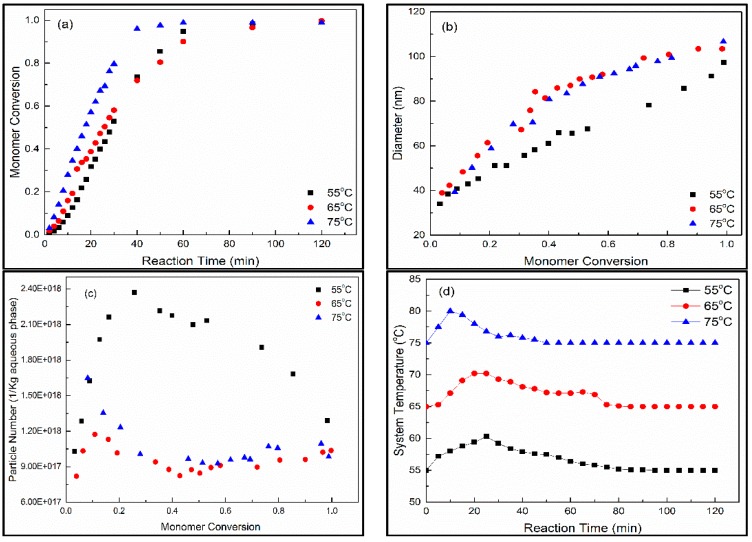
Plots of monomer conversion (**a**); particle size (**b**); number (**c**); and system temperature (**d**) against reaction time (or monomer conversion) for the emulsion polymerization of styrene using methanol solution (20/80 *w*/*w*) as the polymerization medium and various reaction temperatures (55, 65, and 75 °C).

The evolution of the monomer conversion, average particle size, number and system temperature as a function of reaction time (or monomer conversion) of KPS system at different initiation reaction temperatures is shown in Figure 5. The rapid increase in average particle size and the decrease in the particle number confirmed the particle coagulation behavior during the polymerization process. The initial time of the decrease in the particle number showed that the starting time of particle coagulation advanced to the nucleation period with increasing initiation reaction temperature. As the reaction temperature increased, the total polymerization reaction time shortened, as shown in Figure 5a. In addition, the time corresponding to the highest system temperature shifted to 10 min from 24 min with increasing reaction temperature, as expected.

### 3.2. AIBN and AIBA Initiator Systems

**Figure 6:**
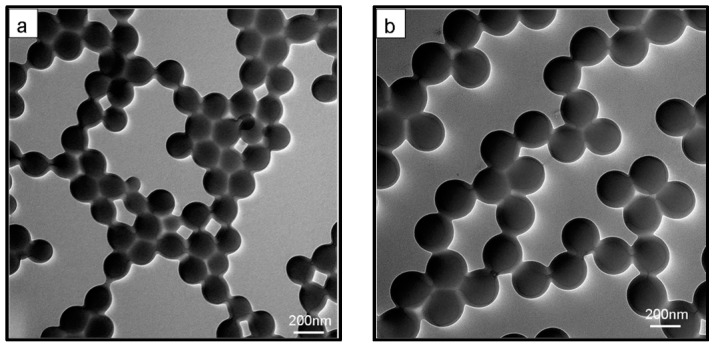
TEM micrographs of the final particles prepared by emulsion polymerization of styrene using methanol solution (20/80 *w*/*w*) as the polymerization medium and various initiator systems: (**a**) AIBN and (**b**) AIBA. The TEM micrograph of the final particles prepared by emulsion polymerization of styrene initiated by KPS is shown in Figure 1b.

As mentioned above, other type of initiators, such as oil-soluble AIBN and cationic AIBA, could also be used to initiate emulsion polymerization reactions. Differing from the conventional KPS initiator, the oil-soluble initiator AIBN scarcely dissolves in aqueous media during the polymerization process. From the AIBN solubility view, the initiator decomposition reaction should be carried out in these monomer droplets or swollen micelles according to a similar bulk method. However, the small reaction volume of the swollen-micelles (or monomer droplets) made the initiator radicals easy to recombine, further limiting the initiation reactions that occurred in monomer droplets [33,34,35,36,37]. Thus, the kinetic model of emulsion polymerization using oil-soluble AIBN was closer to the zero-one model, rather than the pseudo-bulk model [48,49,50]. Figure 6 shows that the TEM images of ultimate latex particles prepared using AIBN and AIBA initiators at 0.6 wt % concentration. The results indicated that the latex particles using AIBN and AIBA were much larger than those using KPS initiator system. The average particle size attained were 141.2 and 224.6 nm with AIBN and AIBA, respectively.

**Figure 7:**
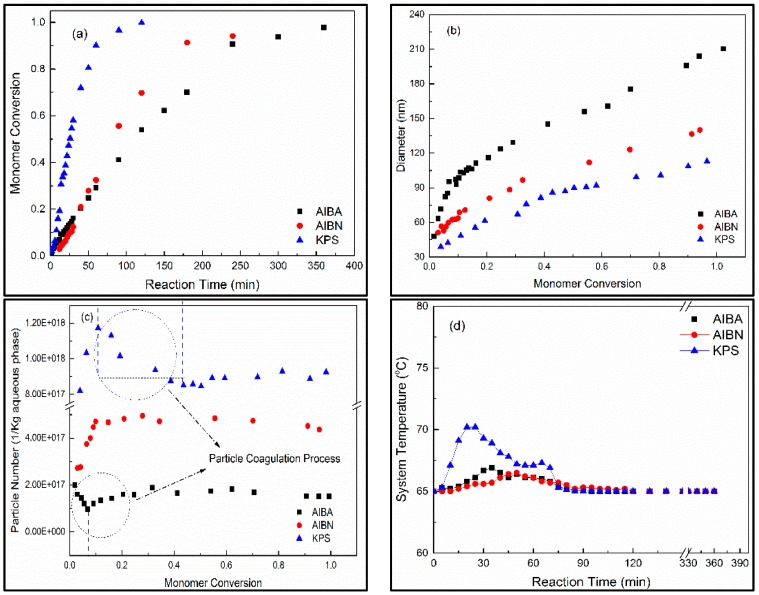
Plots of the monomer conversion (**a**); particle size (**b**); particle number (**c**); and system temperature (**d**) against monomer conversion (or reaction time) for the emulsion polymerization of styrene using methanol solution (20/80 *w*/*w*) as the polymerization medium and various types of initiator.

The curves of monomer conversion, particle size, particle number, and system temperature *vs.* monomer conversion (or reaction time) shown in Figure 7 indicate that particle coagulation was scarcely observed for the AIBN system because no decrease in particle number was observed in Figure 7c. In contrast to AIBN system, an obvious particle coagulation process was observed in the AIBA system, as shown in Figure 7c. Figure 7a shows that the KPS initiator system only needed ~120 min reaction time when the monomer conversion reached 1, even though particle coagulation occurred. However, the systems initiated by AIBN and AIBA needed 240 and 300 min, respectively. The curves of the particle number *vs.* monomer conversion indicate that the initial particle numbers of the AIBA and the AIBN systems were much smaller than the KPS one. The evolution of the system temperature inside the reactor initiated by AIBA and AIBN is also traced, as shown in Figure 7d. However, the variation in the system temperature was difficult to observe because of the slow polymerization rate. In the whole polymerization process, the maximum system temperatures for AIBA and AIBN were slightly higher than the reaction temperature, 67.5 and 67.2 °C, respectively.

## 4. Discussion

Based on these experimental results, the effect of initiator systems on particle coagulation and particle size distribution is discussed.

The extent of particle coagulation was indeed controlled by adjusting the initiator system, especially by using ionic initiators such as KPS and AIBA. The addition of KPS not only increased the primary radical concentration in aqueous media, but also increased the ionic strength. The increase in the primary radical concentration increased the probability of particle collision, and further promoted particle coagulation occurred. This process could be expressed by the modified Von Smoluchowski equation [51]:

(2) $$B\left(i,\;j\right)=f\left(\gamma \right)N\left(i\right)N\left(j\right){\left[d_{p}\left(i\right)+d_{p}\left(j\right)\right]}^{3}$$

where *B*(i,j) and *f*(γ) are the number of collisions between particles i and j class and constant, respectively. Because of the increase in the primary particle number, the B(i,j) directly increased, thus enhancing the extent of particle coagulation. This process was controlled by the kinetics, as described in the Introduction Section. As the particle coagulation was carried out, *N*(i) and *N*(j) decreased, thus the collision frequency between particles i and j class decreased. Meanwhile, the larger particles obtained by the particle coagulation between particles i and j class was difficult to aggregate unless the number of larger particles attained a critical value. On the other hand, KPS and AIBA initiator could also be considered as the electrolyte in polymerization recipe, and the addition of ionic initiator enhanced the ionic strength. Furthermore, the thickness of electrical double layer surrounding the particle surface was compressed, which decreased the particle stability and increased the extent of particle coagulation [25]. The oil-soluble AIBN initiator was not ionic, therefore, the addition of AIBN scarcely affected the thickness of electrical double layer, and, furthermore, particle coagulation was not obvious. Meanwhile, when the oil-soluble AIBN was used as initiator, the vast majority of the initiator dissolved in monomer droplets, monomer-swollen micelles, and polymer particles, and only a small quantity of initiator dissolved in the aqueous phase, which initiated the polymerization reaction. Because the pairs of radicals produced within a volume as small as a monomer-swollen latex particle or a monomer-swollen micelle are easily recombined, the free radicals produced from the fraction of initiator dissolved in the aqueous phase are responsible for particle formation and growth. Therefore, the deactivation of the oil-soluble initiator plays a significant role in determining the initial particle number and the rate of the polymerization reaction. Nomura *et al.* indicated that the efficiency of the oil-soluble initiator was only 1/9 of that of KPS in pure water solvent [52]. In the oil-soluble initiator system, the relationship between particle number and initiator was similar to conventional emulsion initiated by KPS system, indicating that the particle number decreased with decreasing initiator concentration. The deactivation of the AIBN is equivalent to the decrease in initiator concentration. As a result, the initial particle number of the oil-soluble initiator was much smaller than that initiated by KPS. The smaller the particle number was, the smaller the frequency of particle collision achieved, which limited the occurrence of particle coagulation. Notably, in the AIBA initiator system, the initial particle number was much smaller than that of the KPS system. This might be attributed to the *in situ* charge neutralization. The polymer initiated AIBA possessed cationic chain ends, which shielded the anionic charge of surfactant molecules SDS adsorbed on the particle surface. As a result, particle coagulation occurred in the early polymerization period. Even though particle coagulation occurred in particle nucleation period, the slight increase in particle number was also observed in this period (Figure 7c). The increase in particle number might be attributed to the new particle formed in aqueous phase. The particle number would increase when the rate of particle formed in aqueous phase is larger than the rate of particle coagulation [30,32]. As the polymerization reaction was carried out, the particle number gradually attained a constant value because a balance between particle nucleation and coagulation was obtained. The earlier the particle coagulation, the narrower the particle size distribution of ultimate latex particles. The curves of particle number *vs.* monomer conversion confirm that the time of particle coagulation could affect the polydispersity of the final latex particles because of the competitive growth mechanism [53]. If the particle coagulation occurred in the early nucleation period, the latex particles were completely swollen by styrene monomer because of the presence of monomer droplets. Thus, the particles were practically soft, leading to the primary coagulation of latex particles easily merging into the larger one [25]. The process of particle coagulation was controlled by the kinetic factors such as the particle collision. In the next particle growth period, the larger particle size had a smaller ratio of surface and volume and, furthermore, had a relatively slow rate of capturing monomer radicals, resulting in a much slower growth rate of larger particles than the smaller ones [6]. As a result, the narrowly dispersed latex particles were obtained in the presence of particle coagulation.

From the thermodynamic view, the initiator systems also affect the electrostatic force among the particles themselves. For instance, the cationic AIBA initiator systems decomposed into initiator radicals, and the initiator radicals reacted with styrene monomer dissolved in aqueous media and formed the monomer radicals with cationic initiator ends. These monomer radicals were captured by the swollen micelles formed by SDS molecules, and further shielded the negative charge of the surfactant SDS molecules adsorbed on the particle surface, and decreased the electrostatic repulsion among the particle themselves. As a result, *in situ* charge neutralization process occurred, further increasing the extent of the particle coagulation, and promoting particle coagulation that occurred in the early nucleation period.

## 5. Conclusions

In conclusion, we demonstrated the effect of initiator systems on the particle coagulation and particle size distribution of ultimate latex particles. The change in the initiator systems not only affects the extent of the particle coagulation, but also determines the time of particle coagulation. In the anionic KPS system, with the increase in the initiator concentration, the extent of particle coagulation was enhanced, resulting in obtaining larger size and narrowly dispersed latex particles. The cationic AIBA initiator played a significant role in determining the time of particle coagulation. The positive charges derived from AIBA chain ends shield the negative charge of surfactant SDS molecules adsorbed on the particle surface, leading to *in situ* charge neutralization, further enhancing the extent of particle coagulation and advancing the time of particle coagulation. The kinetics of oil-soluble AIBN systems seems similar to that of a conventional polymerization because of the deactivation of the oil-soluble initiator. Thus, combined with our previous reports on the particle coagulation, a systematic method for controlling the particle size distribution in the presence of particle coagulation was achieved.

## Figures and Tables

**Table 1:** The polymerization fundamental recipe for investigating the effect of initiator systems on particle coagulation.

Formulation parameter	Reagents	Quantity (g)
Monomer	St	100
Surfactant	SDS	1.5
Initiator	KPS/AIBN/AIBA	Variable
Electrolyte	K_2_CO_3_	0.6
Co-solvent	methanol	30
